# Association of* PTPN22* Haplotypes (−1123G>C/+1858C>T) with Rheumatoid Arthritis in Western Mexican Population

**DOI:** 10.1155/2017/8753498

**Published:** 2017-01-22

**Authors:** Yeniley Ruiz-Noa, Jorge Ramón Padilla-Gutiérrez, Jorge Hernández-Bello, Claudia Azucena Palafox-Sánchez, Yeminia Valle, Edith Oregón-Romero, Ana Laura Pereira-Suárez, Ana Guilaisne Bernard-Medina, José Francisco Muñoz-Valle

**Affiliations:** ^1^Instituto de Investigación en Ciencias Biomédicas, Centro Universitario de Ciencias de la Salud, Universidad de Guadalajara, Guadalajara, JAL, Mexico; ^2^Servicio de Reumatología, OPD Hospital Civil de Guadalajara “Fray Antonio Alcalde”, Guadalajara, JAL, Mexico

## Abstract

Rheumatoid arthritis (RA) is an autoimmune disease characterized by the presence of antibodies against cyclic citrullinated peptide (anti-CCP), a consequence of the breakdown of immune tolerance. The lymphoid tyrosine phosphatase (Lyp) protein has significant effects on maintenance of peripheral immune tolerance. Two polymorphic variants (−1123G>C and +1858C>T) at* PTPN22 *gene that encodes this protein have been associated with autoimmune disorders and found in strong linkage disequilibrium in Caucasian population. We evaluated whether* PTPN22* haplotypes (−1123G>C/+1858C>T) are associated with anti-CCP antibodies, as well as susceptibility to RA in a Western Mexican population. A total of 315 RA patients and 315 control subjects (CS) were included. The polymorphisms were genotyped by PCR-RFLP and the anti-CCP antibodies were determined by ELISA. The* PTPN22* polymorphisms were in strong linkage disequilibrium (D′ = 1.00 in CS). The susceptibility haplotype CT was significantly more frequent in RA patients than in CS (OR 2.18, 95% CI 1.15–4.16, *p* = 0.01). No association between haplotypes and anti-CCP antibodies levels was observed. In conclusion, this study confirmed that −1123G>C and +1858C>T* PTPN22* polymorphisms are in strong linkage disequilibrium and the CT haplotype is a susceptibility marker to RA in Western Mexico. However, the* PTPN22* haplotypes are not associated with anti-CCP antibodies.

## 1. Introduction

Rheumatoid arthritis (RA) is a complex autoimmune disease, characterized by immune cell-mediated destruction of the joints. Genetic factors appear as major contributors to its pathogenesis [1]. Single nucleotide polymorphisms (SNP) in the protein tyrosine phosphatase nonreceptor type 22 gene* (PTPN22)* encoding the lymphoid protein tyrosine phosphatase LYP [2] were found to be associated with several autoimmune disorders [3–10]. LYP is a potent negative regulator of the lymphocytic activation; it acts at the initial steps of T-cell receptor (TCR) signaling [11].

*PTPN22* +1858C>T SNP (rs2476601) has been consistently associated with RA [12–14]. This SNP consists of a single-base change resulting in a substitution of Arg to Trp in the 620 position at P1 domain of the phosphatase, which plays an important role in the interaction to Csk kinase in order to downregulate the activation signaling pathway, causing by this an hyperresponsive T and B cell phenotype [15].

Nevertheless, in Asian population, the polymorphic variant of this SNP was not found, suggesting that the presence of another variant is associated with the disease. In that study, the authors identified a novel promoter polymorphism, the −1123G>C (rs2488457), which was associated with acute-onset but not slow-onset type 1 diabetes (T1DM) in the Japanese and Korean population [16]. Similar studies in Caucasian and Asian patients revealed a strong association between −1123G>C SNP with RA [17, 18].

The haplotype constructions of both SNPs have been tested in several populations and strong linkage disequilibrium was found between them [16, 19–21]. The CT haplotype that includes the risk alleles from both* PTPN22 *polymorphisms (−1123G>C and +1858C>T) has shown a significant association with T1DM [16, 19, 20], RA [21], and juvenile idiopathic arthritis (JIA) [19].

The most common feature of RA is the presence of antibodies to citrullinated proteins/peptides (ACPAs). ACPAs comprise a group of antibodies that are highly specific for the disease; among them are anti-CCP antibodies. The presence of high levels of anti-CCP at early diagnosis predicts more pronounced radiographic progression, as has been demonstrated by several studies [22–24].

Based on this knowledge, the aim of our study was to investigate the association of* PTPN22* haplotypes (−1123G>C/+1858C>T) with RA and their relationship with anti-CCP antibodies in patients from Western Mexico.

## 2. Material and Methods

### 2.1. RA Patients and Control Subjects

Our study enrolled three hundred fifteen RA patients and the same number of subjects with no medical record of autoimmune or chronic diseases, named as control subjects, all of them with Western Mexican origin. Patients were recruited from the Rheumatology Department of Hospital Civil “Fray Antonio Alcalde,” Guadalajara, Jalisco, Mexico, and they were classified according to the 2010 ACR/EULAR criteria [25]. Clinical and demographic characteristics of this group are shown in Table 1. We observed a female predominance in our RA population (93%); their mean age was 48 ± 14 years, and most patients had disease duration of ten years and mean disease activity score DAS28 of 4.8. All individuals signed the written informed consent and this study was approved by the ethical committee of Universidad de Guadalajara.

### 2.2. *PTPN22* −1123G>C and +1858C>T SNPs Genotyping

Genomic DNA was extracted from peripheral blood cells according to a modified Miller technique. Genotyping was performed using polymerase chain reaction followed by a restriction fragment length polymorphism (PCR-RFLP) technique for the* PTPN22* −1123G>C and +1858C>T polymorphisms. PCR products were digested for one hour at 37°C with appropriate restriction enzymes and then resolved on a 6% polyacrylamide gel.

For the −1123G>C polymorphism a 205 bp fragment was amplified (following our previously reported method) [26] and digested with* Sac I* restriction enzyme (New England Biolabs, Ipswich, MA, USA). The polymorphic allele −1123C was cleaved in two fragments of 183 bp and 22 bp, respectively, while the −1123G allele remained intact. For the +1858C>T SNP we used previously reported conditions [14]. Amplified products of 412 bp were digested with* Xcm I* restriction enzyme (New England Biolabs, Ipswich, MA, USA); only the polymorphic +1858T allele was cleaved into two fragments of 246 bp and 166 bp. Results were confirmed by automatized sequencing of one random sample of each genotype (Applied Biosystems, USA).

### 2.3. Enzyme-Linked Immunoassays

Anti-CCP antibodies were measured in serum samples from RA patients and CS by enzyme-linked immunosorbent assay (ELISA) (Anti-CCP, DIASTAT™, Axis Shield Diagnostics, Dundee, UK). The cut-off level was 5 U/mL for semiquantitative analysis. The immunoassays were performed following the manufacturer's instructions. The sensitivity limit of the assay was 1.04 U/mL.

### 2.4. Statistical Analysis

Hardy-Weinberg equilibrium was determined by a Chi-squared test. Haplotype frequencies were estimated based on expectation–maximization algorithm according to Slatkin and Excoffier [27]. Linkage disequilibrium (LD) was expressed as Lewontin's coefficient (D′) using Arlequin software version 3.0. Haplotype frequency comparisons between RA patients and CS were performed using odds ratios (OR) with 95% confidence intervals (CI) by Fisher's exact test.* p* values < 0.05 refer to a two-sided test which was considered statistically significant. For other comparisons we used Mann–Whitney* U* test, SPSS 18.0 software (SPSS Inc., Chicago, IL, USA).

## 3. Results

### 3.1. Clinical and Demographic Features

Clinical and demographic data of the RA patients and control subjects (CS) groups are shown in Table 1. The RA patients consisted predominantly of women of 48 (18–82) years with a long-term disease evolution (median ten years), while the median of year in CS was 36 years. The RA presented a condition with moderate clinical activity defined by the DAS28 index (4.8 ± 1.4 score) and some functional disability degree, according to Spanish HAQ-DI index (0.76 ± 0.67 score). Furthermore, 83% of total patients were positive for anti-CCP antibodies, and 84% were positive for RF. None of the control subjects are positive to anti-CCP antibodies, and only 5% are positive for RF.

### 3.2. Linkage Disequilibrium and Haplotypes of* PTPN22* Gene

The genotypes and alleles distribution of the* PTPN22* −1123G>C and +1858C>T polymorphisms were in Hardy-Weinberg equilibrium among control population (*p* > 0.05). Haplotype constructions of two SNPs revealed three of four possible haplotypes (with a frequency >1%); GC, CC, and CT haplotypes. The linkage disequilibrium analysis showed the strongest linkage between −1123G>C and +1858C>T* PTPN22 *polymorphisms (D′ = 1, *p* = 0.001) calculated for CS. The* PTPN22* susceptibility haplotype (−1123C/+1858T or CT), which included the polymorphic alleles from both SNPs, was more frequent in RA patients than in CS (OR 2.18, 95% CI 1.15–4.16, *p* = 0.01) (Table 2).

### 3.3. Association of* PTPN22* Haplotypes with Anti-CCP Antibodies

**Figure 1:**
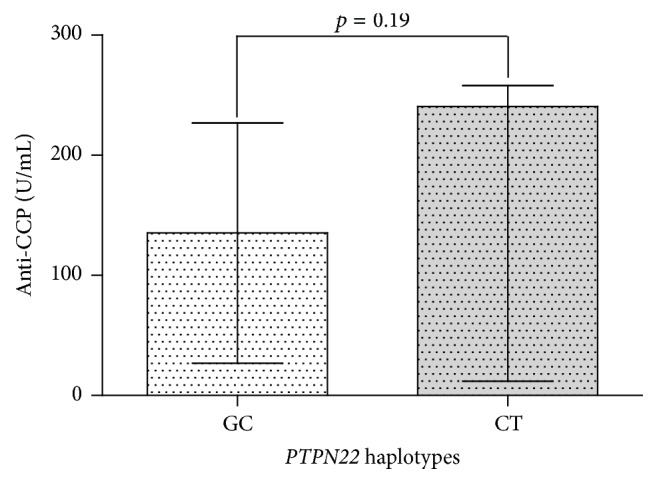
Levels of anti-CCP autoantibodies according to homozygous haplotypes of the* PTPN22* −1123G>C and +1858C>T SNPs. GC nonrisk haplotype and CT risk haplotype. The medians and interquartile ranges of the parameters are represented as a black line. Levels of anti-CCP are given in international units (U/mL) according to the Axis Shield Company.

Furthermore, we compared anti-CCP antibodies levels between RA patients homozygous for* PTPN22* susceptibility haplotype CT and homozygous carriers of (−1123G/+1858C or GC) nonsusceptibility* PTPN22* haplotype, which included the wild alleles from both SNPs. Our results did not show statistical differences among levels of anti-CCP antibodies among those patients (Figure 1).

Later, we selected anti-CCP positive RA patients and stratified them according to the antibodies levels. Patients who had anti-CCP antibodies levels higher than the upper limit of normal (ULN) laboratory assay, but less or equal to 3 times this value, were considered as low positive and patients with anti-CCP antibodies values that are higher than 3 times ULN were considered high positive (according to 2010 ACR/EULAR criteria) [25]. These groups were compared according to the presence of CT susceptibility or GC nonsusceptibility* PTPN22* haplotypes (in individuals homozygous for both SNPs), to determine whether haplotypes influence the anti-CCP antibodies status. In our comparison, we did not find significant differences between carriers of susceptibility or nonsusceptibility haplotypes (Table 3). The relationship between anti-CCP levels and these haplotypes was also compared among RA patients with or without a history of smoking habit, but we did not find a significant correlation (data not shown).

## 4. Discussion

Haplotype frequencies and D′ values in our study indicate strong linkage disequilibrium between both* PTPN22* SNPs (D′ = 1.00) which is in concordant with previous reports from Caucasian [16, 19, 21], Japanese [16], Norwegian [21], and Polish [20] populations. The extent of linkage disequilibrium found by these authors was very similar to ours (with* D*′ values in CS: 0.99, 0.97, 0.99, and 0.97, resp.) [16, 19, 20].

The frequency distribution analysis revealed a significant increase in the susceptibility haplotype comprised by the risk alleles (−1123C and +1858T) of both SNPs, compared among RA patients and controls subjects. There is only one previous study in which the association between haplotypes formed by the* PTPN22* −1123G>C and +1858C>T polymorphisms and RA gene was established [21]. In this report, the authors demonstrated an association between the CT haplotype consisting of the risk alleles of both polymorphisms and RA (OR 1.54, 95% CI 1.23–1.93, *p* = 0.0002). Our results agree with this work, as haplotype association between CT and RA susceptibility was established.

Altogether, these results confirm the direct involvement of* PTPN22* gene independently of HLA* locus* in the pathogenesis of autoimmune diseases. However, considering the limited effect that has shown individually the −1123 G>C SNP in some reports, it is evident that the potential effects of the CT haplotype are mainly due to +1858 C>T* PTPN22 *SNP [28]. Thus, that is the reason why there was no evidence for an independent effect at +1858 C>T in some studies [19, 29, 30].

In established comparisons between the levels of anti-CCP antibodies between individuals carrying the susceptibility haplotype CT with those who had nonsusceptibility haplotype GC, we did not find any significant difference (*p* = 0.196). However, there is a marked tendency in patients with CT haplotype to have higher anti-CCP levels (median value of 240.5 U/mL) than those obtained for patients carrying GC haplotype (median value of 83.5 U/mL). These negative results can be due to a low frequency of the individuals homozygous to the risk haplotype CT in our population. Another important point to consider is the treatment and the evolution time of these patients, since they can modify the levels of anti-CCP antibodies; so it would be interesting to evaluate the outcome in patients with early RA, if possible, prior to treatment with disease modifying drugs (DMARDs).

Anti-CCP status was defined by 2010 ACR/EULAR criteria as negative, low, or high positive anti-CCP levels [25]. Later, we stratified seropositive RA patients group according to this classification and did not find statistical differences (*p* = 0.873) between subjects carrying the CT risk haplotype compared to patients carrying GC haplotype, which indicates that CT risk haplotype did not predispose to high positive or any particular anti-CCP status; this observation remains constant even in smokers.

It is important to note that until now there are no studies that compare status or levels of anti-CCP antibodies with* PTPN22* haplotypes in rheumatoid arthritis patients; in this respect, our study is the first of its type.

In conclusion, −1123G>C and +1858C>T* PTPN22* SNPs are in strong linkage disequilibrium and (−1123C/+1858T) haplotype, which comprises polymorphic variants from both polymorphisms, is associated with susceptibility to RA but is not associated with status or higher levels of anti-CCP antibodies in a Western Mexican population. However, further studies are needed to elucidate the relationship between the risk haplotype CT and anti-CCP antibodies.

## Figures and Tables

**Table 1:** Demographic and clinical characteristics of RA patients.

Variables	RA (*n* = 315)	CS (*n* = 315)
*Demographic and clinic characteristics*		
Age (years)^a^	48 (18–82)	36 (18–74)
Sex% (*n*)^b^		
Males	7 (23)	40 (127)
Females	93 (292)	60 (188)
Smokers^b^	29 (71)	9 (29)
*Disease status*		
Duration of disease (years)^a^	10 (0.1–52)	—
DAS28^c^	4.8 ± 1.4	—
Spanish HAQ-DI score^a^	0.76 (0–2.28)	—
*Autoantibodies*		
Rheumatoid factor (IU/mL)^a^	178 (15–717)	15 (0–62)
Negative (<20 UI/mL)^b^	16 (50)	95 (300)
Low positive (20–59.9 UI/mL)^b^	21 (65)	5 (15)
High positive (≥60 UI/mL)^b^	63 (200)	0 (0)
Anti-CCP (U/mL)^a^	119 (2–460)	1.5 (0.9–2.5)
Negative (<5 U/mL)^b^	17 (52)	100 (315)
Low positive (5.1–14.9 U/mL)^b^	9 (27)	0 (0)
High positive (≥15 U/mL)^b^	74 (236)	0 (0)

^a^Data presented in median (p25–p75). ^b^Data provided in percentage and *n*. ^c^Data provided in mean ± SD. RA: rheumatoid arthritis; CS: control subject; Anti-CCP: anticyclic citrullinated peptide antibody; DAS28: disease activity score 28; Spanish HAQ-DI: Spanish version of the Health Assessment Questionnaire Disability Index.

**Table 2:** Association analyses of the *PTPN22* haplotypes constructed for the −1123G>C and +1858C>T SNPs in RA patients and CS.

Haplotypes −1123G>C/+1858C>T	RA (2^*n*^ = 630)%	CS (2^*n*^ = 630)%	OR	(95% CI)	*p*
GC	66.26	63.33	NA	NA	NA
CC	28.18	34.44	0.78	(0.62–0.99)	0.05
CT	5.15	2.22	**2.18**	** (1.15–4.16)**	**0.01**
GT	0.40	0	6.69	(0.34–130.09)	0.25

Values represent a frequency of cases with respect to total number of haplotypes, for RA patients and control subject. SNP: single nucleotide polymorphism; RA: rheumatoid arthritis; CS: control subjects; OR: odds ratios; CI: confidence intervals; *p* < 0.05 level of significance for Fisher exact test; NA: not applicable.

**Table 3:** Frequencies of −1123G>C and +1858C>T *PTPN22* haplotypes in RA seropositive patients stratified according to anti-CCP antibodies status.

	GC% (*n*)	CT% (*n*)	OR	95% CI	*p *value
Anti-CCP low positive^a^	3.9 (5)	0 (0)	—	—	—
Anti-CCP high positive^b^	82.8 (106)	100 (3)	1.028	(0.996–1.061)	0.873

^a^Referred to antibodies values that are > than the upper limit of normal (ULN) and ≤ 3 times the ULN for laboratory assay; ^b^referred to antibodies values that are > than 3 times ULN for laboratory assay (according to ACR/EULAR 2010 criteria). OR: odds ratio; 95% CI: confidence interval; anti-CCP: anti-cyclic citrullinated peptide; Ab: antibody; RA: rheumatoid arthritis. The haplotypes include only homozygous individuals for each polymorphism.
